# (3a*R*,6*S*,7a*R*)-7a-Bromo-2-[(4-methyl­phen­yl)sulfon­yl]-1,2,3,6,7,7a-hexa­hydro-3a,6-ep­oxy­isoindole

**DOI:** 10.1107/S1600536811010750

**Published:** 2011-03-31

**Authors:** Başak Koşar, Aydın Demircan, Hakan Arslan, Orhan Büyükgüngör

**Affiliations:** a Department of Science Education, Faculty of Education, Sinop University, 57100-Sinop, Turkey; b Department of Chemistry, Faculty of Arts and Sciences, Nigde University, 51240-Nigde, Turkey; c Department of Chemistry, Faculty of Arts and Science, Mersin University, 33343-Mersin, Turkey; d Department of Physics, Arts and Sciences Faculty, Ondokuz Mayıs University, 55139-Samsun, Turkey

## Abstract

In the title compound, C_15_H_16_BrNO_3_S, the boat form of the six-membered ring is almost symmetrical with respect to the ep­oxy bridge. The two five-membered rings generated by the ep­oxy bridge of the six-membered ring adopt envelope conformations, whereas the N-containing five-membered ring adopts a twisted conformation. In the crystal, mol­ecules are linked by C—H⋯O hydrogen bonds.

## Related literature

For general background to intra­molecular Diels–Alder reactions and heteroaromatic Diels–Alder reactions, see: Dell (1998 ▶); Kappe *et al.* (1997 ▶); Arai *et al.* (2010 ▶); Lohse & Hsung (2009 ▶). For related structures, see: Koşar *et al.* (2006 ▶, 2007*a* ▶,*b* ▶). For the synthesis of the title compound and related compounds, see: Carlini *et al.* (1997 ▶); Hart *et al.* (1997 ▶); Shing *et al.* (1996 ▶); Karaarslan *et al.* (2007 ▶); Pontén & Magnusson (1997 ▶); Demircan *et al.* (2006 ▶); Arslan & Demircan (2008 ▶); Demircan & Parsons (1998 ▶). For puckering analysis, see: Cremer & Pople (1975 ▶).
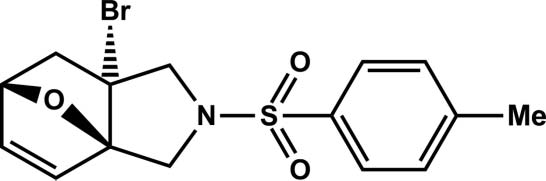


## Experimental

### 

#### Crystal data

C_15_H_16_BrNO_3_S
*M* *_r_* = 370.26
Monoclinic, 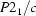

*a* = 16.5136 (6) Å
*b* = 6.2186 (3) Å
*c* = 16.3487 (7) Å
β = 113.802 (3)°
*V* = 1536.07 (12) Å^3^
*Z* = 4
Mo *K*α radiation
μ = 2.82 mm^−1^
*T* = 296 K
0.58 × 0.44 × 0.31 mm

#### Data collection

STOE IPDS 2 CCD diffractometer
Absorption correction: integration (*X-RED32*; Stoe & Cie, 2002 ▶) *T*_min_ = 0.310, *T*_max_ = 0.495
7305 measured reflections
3163 independent reflections
2353 reflections with *I* > 2σ(*I*)
*R*_int_ = 0.042

#### Refinement

*R*[*F* ^2^ > 2σ(*F* ^2^)] = 0.048
*wR*(*F* ^2^) = 0.126
*S* = 1.07
3163 reflections
190 parameters
H-atom parameters constrained
Δρ_max_ = 0.40 e Å^−3^
Δρ_min_ = −0.44 e Å^−3^

### 

Data collection: *X-AREA* (Stoe & Cie, 2002 ▶); cell refinement: *X-AREA*; data reduction: *X-RED32* (Stoe & Cie, 2002 ▶); program(s) used to solve structure: *SHELXS97* (Sheldrick, 2008 ▶); program(s) used to refine structure: *SHELXL97* (Sheldrick, 2008 ▶); molecular graphics: *OLEX2* (Dolomanov *et al.*, 2009 ▶); software used to prepare material for publication: *SHELXTL* (Sheldrick, 2008 ▶), *OLEX2*, *publCIF* (Westrip, 2010 ▶) and *Mercury* (Macrae *et al.*, 2006 ▶).

## Supplementary Material

Crystal structure: contains datablocks global, I. DOI: 10.1107/S1600536811010750/zl2354sup1.cif

Structure factors: contains datablocks I. DOI: 10.1107/S1600536811010750/zl2354Isup2.hkl

Additional supplementary materials:  crystallographic information; 3D view; checkCIF report

## Figures and Tables

**Table 1:** Hydrogen-bond geometry (Å, °)

*D*—H⋯*A*	*D*—H	H⋯*A*	*D*⋯*A*	*D*—H⋯*A*
C6—H6*A*⋯O1^i^	0.97	2.50	3.382 (6)	151

Symmetry code: (i) 
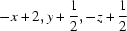
.

**Fig. 1.**
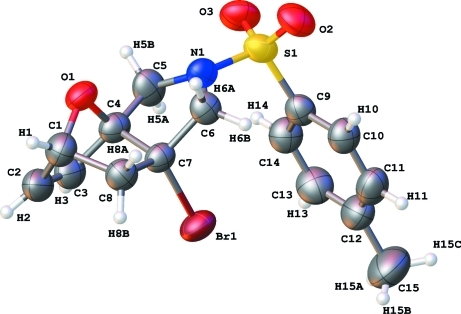
The molecular structure of the title compound, with displacement ellipsoids drawn at the 30% probability level.

**Fig. 2.**
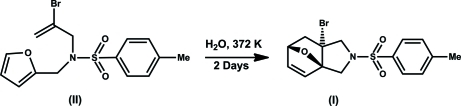
Synthesis of the title compound.

**Fig. 3.**
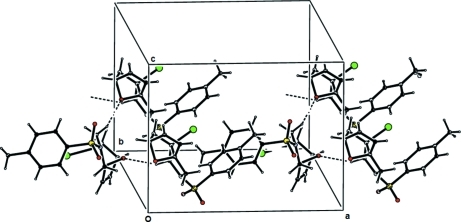
A packing diagram of the title compound. Dashed lines indicate the O—H···O intermolecular hydrogen bonds.

## supplementary crystallographic information

### Comment

Thermal intramolecular [4 + 2] type cycloaddition processes, or intramolecular Diels Alder (IMDA) reactions, have been a highly useful tool for the construction of many cycloaddition products (Dell, 1998). The IMDA reaction is especially useful for asymmetrical syntheses towards natural products such as, (±)-xestoquinone (Carlini *et al.*, 1997), (+)-himbelive (Hart *et al.*, 1997) and (S)-(±)-carvone (Shing *et al.*, 1996). In this context, the use of heteroaromatic compounds has been gaining popularity (Kappe *et al.*, 1997). IMDA reactions with furan derivatives, called IMDAF, have been widely used for the construction of some new molecules (Karaarslan *et al.*, 2007; Pontén & Magnusson, 1997; Demircan *et al.*, 2006; Koşar *et al.*, 2007*a*; Koşar *et al.*, 2007*b*; Arslan & Demircan, 2008; Demircan & Parsons, 1998; Koşar *et al.*, 2006). These compounds have been used as strategic intermediates in combinatorial synthesis.

In view of a recent literature research (Arai *et al.*, 2010; Lohse & Hsung, 2009), we would like to report here a thermal IMDAF reaction of an alkenyl furan with a nitrogen linked chain which undergoes intramolecular cycloaddition upon heating to 371 K for two days (Fig. 1 and 2). The product of the intramolecular thermal cycloaddition reaction of compound (I) was characterized by ^1^H NMR, ^13^C NMR, FT-IR, MS, elemental analysis and X-ray single crystal diffraction studies. Despite of the presence of three new stereocenters in the product molecules only one pair of mirror symmetric enatiomers was formed in the reaction, i.e. the intramolecular thermal cycloaddition of II to I is diastereoselective under the chosen reaction conditions.

Related with the above mentioned reaction, we presented here the crystal structure of the title compound, C_15_H_16_BrNO_3_S. Fig. 1 shows the molecular structure of the title compound, I. The pyrrolidine (C4/C5/N1/C6/C7) ring adopts a twisted conformation with a total puckering parameter *Q*_T_ value of 0.329 (5) Å (Cremer & Pople, 1975). The tetrahydrofuran (O1/C1-C4) and bromo-attached tetrahydrofuran (O1/C4/C7/C8/C1) rings adopt envelope conformations with total puckering parameters of 0.514 (5) and 0.627 (5) Å, respectively.

The title compound displays an intramolecular hydrogen bond between atoms C10 and O2 and the crystal structure is stabilized by weak van der Waals interactions and a weak intermolecular hydrogen bond, C6—H6a···O1 in a three dimensional network (Fig. 3). Geometrical parameters of the intra- and intermolecular H-bonds are listed in Table 1.

### Experimental

*N*-(2-Bromoprop-2-en-1-yl)-4-methyl-*N*-[(5-methyl-2-furyl)methyl]benzenesulfonamide, II, (1 g, 2.7 mmol) was stirred and heated under reflux in water (25 mL) at 372 K for two days (Fig. 2). The mixture was poured into ethyl acetate (25 mL) and the aqueous phase was washed with excess ethyl acetate (2 x 25 mL). The combined organic phases were dried over magnesium sulphate and filtered off. The solvent was then removed under reduced pressure. The residue was subjected to flash column chromatography (*R*_f _(Hexane:Ethyl acetate = 7:3): 0.47) to afford I (0.7 g, 70 % yield) as yellow crystals. M. p.: 396-398 K, ν_max _(Thin film) / cm^-1^: 2932 (s, CH), 2161 (m, SO), 1977 (m, S=O), 1453, 1159, 1065 (s, C-O). δ_H_ (400 MHz, CDCl_3_): 7,67 (d, 2H, *J* = 8 Hz), 7.24 (d, 2H, *J =* 8 Hz, H10-H13), 6.40 (dd, 1H, *J =* 5.6 Hz, *J =* 1.8 Hz, AB), 6.34 (d, 1H, *J =* 5.6 Hz, AB), 4.95 (dd, 1H, *J =* 4.5 Hz, *J =* 1.8 Hz), 4.06 (d, 1H, *J =* 12 Hz), 4.01 (d, 1H, *J =* 12 Hz), 3.67 (d, 1H, *J =* 12 Hz), 3.43 (d, 1H, *J =* 12 Hz), 2.39 (dd, 1H, *J =* 4.5 Hz, *J =* 12 Hz), 2.36 (s, 3H), 1.63 (d, 1H, *J =* 12 Hz). δ_C_ (100 MHz, CDCl_3_): 143.8, 137.2, 134.7, 134.5, 129.9 (2 x C), 127.7 (2 x C), 97.2, 81.0, 64.0, 63.3, 47.5, 41.4, 21.7. m/z (70 eV, EI): 371,00 [M^+^(^81^Br), 42%)], 369, [M^+^(^79^Br, 42%)], 216,00 [M^+^(^81^Br)-Ts, 100%], 214 [M^+^(^79^Br)-H+Ts, 100%]. Elemental Analysis (C_15_H_16_BrNO_3_S): % Calculated (Found): C, 48.66 (48.72); H, 4.36 (4.39); N, 3.78 (3.74).

### Refinement

H atoms were positioned geometrically and treated using a riding model, fixing the bond lengths at 0.96, 0.97, 0.98 and 0.93 Å for CH_3_, CH_2_, CH and CH (aromatic), respectively. The displacement parameters of the H atoms were constrained with *U*_iso_(H) = 1.2*U*_eq_ (aromatic, methylene or methine C) or 1.5*U*_eq_ (methyl C).

### Crystal data

**Table:**

C_15_H_16_BrNO_3_S	*F*(000) = 752
*M**_r_* = 370.26	*D*_x_ = 1.601 Mg m^−^^3^
Monoclinic, *P*2_1_/*c*	Mo *K*α radiation, λ = 0.71069 Å
Hall symbol: -P 2ybc	Cell parameters from 10108 reflections
*a* = 16.5136 (6) Å	θ = 1.5–28.0°
*b* = 6.2186 (3) Å	µ = 2.82 mm^−^^1^
*c* = 16.3487 (7) Å	*T* = 296 K
β = 113.802 (3)°	Prism, colourless
*V* = 1536.07 (12) Å^3^	0.58 × 0.44 × 0.31 mm
*Z* = 4	

### Data collection

**Table:**

STOE IPDS 2 CCD diffractometer	3163 independent reflections
Radiation source: fine-focus sealed tube	2353 reflections with *I* > 2σ(*I*)
plane graphite	*R*_int_ = 0.042
Detector resolution: 6.67 pixels mm^-1^	θ_max_ = 26.5°, θ_min_ = 2.5°
rotation method scans	*h* = −20→20
Absorption correction: integration (*X-RED32*; Stoe & Cie, 2002)	*k* = −7→7
*T*_min_ = 0.310, *T*_max_ = 0.495	*l* = −20→20
7305 measured reflections	

### Refinement

**Table:**

Refinement on *F*^2^	Primary atom site location: structure-invariant direct methods
Least-squares matrix: full	Secondary atom site location: difference Fourier map
*R*[*F*^2^ > 2σ(*F*^2^)] = 0.048	Hydrogen site location: inferred from neighbouring sites
*wR*(*F*^2^) = 0.126	H-atom parameters constrained
*S* = 1.07	*w* = 1/[σ^2^(*F*_o_^2^) + (0.0617*P*)^2^ + 0.5082*P*] where *P* = (*F*_o_^2^ + 2*F*_c_^2^)/3
3163 reflections	(Δ/σ)_max_ < 0.001
190 parameters	Δρ_max_ = 0.40 e Å^−^^3^
0 restraints	Δρ_min_ = −0.44 e Å^−^^3^

### Special details

**Table:**

Geometry. All e.s.d.'s (except the e.s.d. in the dihedral angle between two l.s. planes) are estimated using the full covariance matrix. The cell e.s.d.'s are taken into account individually in the estimation of e.s.d.'s in distances, angles and torsion angles; correlations between e.s.d.'s in cell parameters are only used when they are defined by crystal symmetry. An approximate (isotropic) treatment of cell e.s.d.'s is used for estimating e.s.d.'s involving l.s. planes.
Refinement. Refinement of *F*^2^ against ALL reflections. The weighted *R*-factor *wR* and goodness of fit *S* are based on *F*^2^, conventional *R*-factors *R* are based on *F*, with *F* set to zero for negative *F*^2^. The threshold expression of *F*^2^ > σ(*F*^2^) is used only for calculating *R*-factors(gt) *etc*. and is not relevant to the choice of reflections for refinement. *R*-factors based on *F*^2^ are statistically about twice as large as those based on *F*, and *R*- factors based on ALL data will be even larger.

### Fractional atomic coordinates and isotropic or equivalent isotropic displacement parameters (Å^2^)

**Table:**

	*x*	*y*	*z*	*U*_iso_*/*U*_eq_	
C1	0.9623 (3)	0.6757 (7)	0.0923 (3)	0.0640 (11)	
H1	1.0211	0.7174	0.0966	0.077*	
C2	0.9042 (3)	0.5742 (8)	0.0062 (3)	0.0681 (12)	
H2	0.9082	0.5913	−0.0485	0.082*	
C3	0.8459 (3)	0.4559 (7)	0.0218 (3)	0.0617 (10)	
H3	0.8013	0.3717	−0.0189	0.074*	
C4	0.8668 (3)	0.4857 (6)	0.1192 (3)	0.0537 (9)	
C5	0.8358 (3)	0.3394 (6)	0.1725 (3)	0.0642 (11)	
H5A	0.7765	0.2875	0.1372	0.077*	
H5B	0.8753	0.2172	0.1945	0.077*	
C6	0.8569 (3)	0.7032 (6)	0.2354 (3)	0.0519 (9)	
H6A	0.9170	0.7400	0.2752	0.062*	
H6B	0.8163	0.7984	0.2472	0.062*	
C7	0.8443 (3)	0.7184 (6)	0.1376 (3)	0.0489 (8)	
C8	0.9107 (3)	0.8549 (7)	0.1161 (3)	0.0571 (10)	
H8A	0.9488	0.9380	0.1674	0.069*	
H8B	0.8812	0.9506	0.0660	0.069*	
C9	0.6718 (3)	0.5012 (6)	0.2434 (3)	0.0526 (9)	
C10	0.6473 (3)	0.7028 (7)	0.2619 (3)	0.0617 (10)	
H10	0.6869	0.7852	0.3082	0.074*	
C11	0.5636 (4)	0.7800 (8)	0.2109 (4)	0.0736 (13)	
H11	0.5473	0.9149	0.2236	0.088*	
C12	0.5033 (3)	0.6607 (9)	0.1410 (3)	0.0724 (12)	
C13	0.5291 (3)	0.4605 (9)	0.1249 (3)	0.0725 (12)	
H13	0.4891	0.3777	0.0790	0.087*	
C14	0.6117 (3)	0.3787 (7)	0.1743 (3)	0.0627 (11)	
H14	0.6273	0.2429	0.1617	0.075*	
C15	0.4125 (5)	0.7472 (12)	0.0862 (5)	0.117 (2)	
H15A	0.3802	0.6448	0.0408	0.175*	
H15B	0.4175	0.8799	0.0586	0.175*	
H15C	0.3818	0.7722	0.1242	0.175*	
N1	0.8373 (2)	0.4748 (5)	0.2466 (2)	0.0526 (8)	
O1	0.96199 (18)	0.5098 (4)	0.15645 (19)	0.0589 (7)	
O2	0.8163 (2)	0.5198 (5)	0.38595 (19)	0.0729 (8)	
O3	0.7776 (2)	0.1766 (5)	0.3018 (2)	0.0707 (8)	
S1	0.78021 (8)	0.40698 (16)	0.30290 (6)	0.0559 (3)	
Br1	0.71982 (3)	0.78750 (8)	0.06223 (3)	0.06777 (18)	

### Atomic displacement parameters (Å^2^)

**Table:**

	*U*^11^	*U*^22^	*U*^33^	*U*^12^	*U*^13^	*U*^23^
C1	0.060 (3)	0.068 (3)	0.066 (3)	0.007 (2)	0.028 (2)	0.000 (2)
C2	0.082 (3)	0.067 (3)	0.057 (2)	0.019 (2)	0.030 (2)	−0.001 (2)
C3	0.072 (3)	0.057 (2)	0.055 (2)	0.006 (2)	0.024 (2)	−0.0128 (19)
C4	0.058 (2)	0.043 (2)	0.054 (2)	0.0055 (17)	0.0174 (19)	−0.0063 (17)
C5	0.088 (3)	0.041 (2)	0.069 (3)	−0.003 (2)	0.038 (3)	−0.0124 (19)
C6	0.062 (2)	0.0406 (19)	0.053 (2)	−0.0077 (17)	0.0232 (19)	−0.0103 (17)
C7	0.050 (2)	0.0403 (18)	0.052 (2)	0.0033 (16)	0.0160 (17)	−0.0051 (16)
C8	0.064 (3)	0.047 (2)	0.061 (2)	0.0017 (18)	0.026 (2)	−0.0006 (18)
C9	0.064 (2)	0.044 (2)	0.054 (2)	−0.0029 (18)	0.029 (2)	0.0008 (17)
C10	0.069 (3)	0.052 (2)	0.067 (3)	−0.002 (2)	0.031 (2)	−0.008 (2)
C11	0.084 (3)	0.059 (3)	0.090 (3)	0.013 (2)	0.048 (3)	0.003 (2)
C12	0.063 (3)	0.083 (3)	0.073 (3)	0.007 (2)	0.030 (2)	0.004 (3)
C13	0.066 (3)	0.078 (3)	0.069 (3)	−0.011 (2)	0.022 (2)	−0.009 (2)
C14	0.068 (3)	0.052 (2)	0.069 (3)	−0.006 (2)	0.029 (2)	−0.008 (2)
C15	0.087 (4)	0.140 (6)	0.113 (5)	0.038 (4)	0.029 (4)	0.004 (4)
N1	0.065 (2)	0.0388 (16)	0.0541 (18)	0.0059 (14)	0.0239 (16)	−0.0009 (13)
O1	0.0538 (16)	0.0582 (17)	0.0582 (16)	0.0158 (13)	0.0159 (13)	0.0002 (13)
O2	0.092 (2)	0.077 (2)	0.0429 (14)	−0.0032 (18)	0.0201 (15)	−0.0060 (14)
O3	0.089 (2)	0.0489 (17)	0.0693 (19)	0.0038 (15)	0.0267 (18)	0.0154 (14)
S1	0.0708 (7)	0.0462 (5)	0.0474 (5)	0.0020 (4)	0.0203 (5)	0.0036 (4)
Br1	0.0521 (2)	0.0697 (3)	0.0701 (3)	0.0172 (2)	0.01277 (19)	0.0041 (2)

### Geometric parameters (Å, °)

**Table:**

C1—O1	1.472 (5)	C8—H8A	0.9700
C1—C2	1.487 (7)	C8—H8B	0.9700
C1—C8	1.544 (6)	C9—C10	1.388 (6)
C1—H1	0.9800	C9—C14	1.392 (6)
C2—C3	1.316 (7)	C9—S1	1.757 (4)
C2—H2	0.9300	C10—C11	1.380 (7)
C3—C4	1.498 (5)	C10—H10	0.9300
C3—H3	0.9300	C11—C12	1.390 (8)
C4—O1	1.446 (5)	C11—H11	0.9300
C4—C5	1.487 (6)	C12—C13	1.375 (7)
C4—C7	1.553 (5)	C12—C15	1.502 (8)
C5—N1	1.467 (5)	C13—C14	1.373 (7)
C5—H5A	0.9700	C13—H13	0.9300
C5—H5B	0.9700	C14—H14	0.9300
C6—N1	1.484 (5)	C15—H15A	0.9600
C6—C7	1.530 (5)	C15—H15B	0.9600
C6—H6A	0.9700	C15—H15C	0.9600
C6—H6B	0.9700	N1—S1	1.616 (3)
C7—C8	1.535 (6)	O2—S1	1.427 (3)
C7—Br1	1.971 (4)	O3—S1	1.433 (3)
			
O1—C1—C2	101.0 (4)	C1—C8—H8A	111.7
O1—C1—C8	99.5 (3)	C7—C8—H8B	111.7
C2—C1—C8	109.5 (4)	C1—C8—H8B	111.7
O1—C1—H1	115.0	H8A—C8—H8B	109.5
C2—C1—H1	115.0	C10—C9—C14	119.7 (4)
C8—C1—H1	115.0	C10—C9—S1	120.1 (3)
C3—C2—C1	107.2 (4)	C14—C9—S1	120.1 (3)
C3—C2—H2	126.4	C11—C10—C9	119.5 (4)
C1—C2—H2	126.4	C11—C10—H10	120.3
C2—C3—C4	105.3 (4)	C9—C10—H10	120.3
C2—C3—H3	127.3	C10—C11—C12	121.4 (4)
C4—C3—H3	127.3	C10—C11—H11	119.3
O1—C4—C5	112.9 (3)	C12—C11—H11	119.3
O1—C4—C3	101.9 (3)	C13—C12—C11	117.8 (5)
C5—C4—C3	124.1 (4)	C13—C12—C15	121.5 (5)
O1—C4—C7	97.3 (3)	C11—C12—C15	120.7 (5)
C5—C4—C7	106.9 (3)	C14—C13—C12	122.3 (5)
C3—C4—C7	110.5 (3)	C14—C13—H13	118.8
N1—C5—C4	103.8 (3)	C12—C13—H13	118.8
N1—C5—H5A	111.0	C13—C14—C9	119.3 (4)
C4—C5—H5A	111.0	C13—C14—H14	120.4
N1—C5—H5B	111.0	C9—C14—H14	120.4
C4—C5—H5B	111.0	C12—C15—H15A	109.5
H5A—C5—H5B	109.0	C12—C15—H15B	109.5
N1—C6—C7	104.1 (3)	H15A—C15—H15B	109.5
N1—C6—H6A	110.9	C12—C15—H15C	109.5
C7—C6—H6A	110.9	H15A—C15—H15C	109.5
N1—C6—H6B	110.9	H15B—C15—H15C	109.5
C7—C6—H6B	110.9	C5—N1—C6	112.2 (3)
H6A—C6—H6B	108.9	C5—N1—S1	120.1 (3)
C6—C7—C8	117.7 (3)	C6—N1—S1	121.8 (2)
C6—C7—C4	101.7 (3)	C4—O1—C1	95.1 (3)
C8—C7—C4	102.9 (3)	O2—S1—O3	120.16 (19)
C6—C7—Br1	109.5 (3)	O2—S1—N1	107.28 (19)
C8—C7—Br1	113.4 (3)	O3—S1—N1	105.97 (18)
C4—C7—Br1	110.7 (3)	O2—S1—C9	107.7 (2)
C7—C8—C1	100.1 (3)	O3—S1—C9	107.9 (2)
C7—C8—H8A	111.7	N1—S1—C9	107.18 (17)
			
O1—C1—C2—C3	31.5 (4)	C10—C11—C12—C13	1.0 (7)
C8—C1—C2—C3	−72.9 (5)	C10—C11—C12—C15	−180.0 (5)
C1—C2—C3—C4	0.8 (5)	C11—C12—C13—C14	−1.0 (7)
C2—C3—C4—O1	−33.6 (4)	C15—C12—C13—C14	−180.0 (5)
C2—C3—C4—C5	−162.1 (4)	C12—C13—C14—C9	0.2 (7)
C2—C3—C4—C7	68.9 (4)	C10—C9—C14—C13	0.6 (6)
O1—C4—C5—N1	80.2 (4)	S1—C9—C14—C13	−176.0 (3)
C3—C4—C5—N1	−156.0 (4)	C4—C5—N1—C6	7.1 (5)
C7—C4—C5—N1	−25.6 (4)	C4—C5—N1—S1	160.4 (3)
N1—C6—C7—C8	−139.8 (3)	C7—C6—N1—C5	14.3 (5)
N1—C6—C7—C4	−28.3 (4)	C7—C6—N1—S1	−138.6 (3)
N1—C6—C7—Br1	88.8 (3)	C5—C4—O1—C1	−174.3 (3)
O1—C4—C7—C6	−82.6 (3)	C3—C4—O1—C1	50.3 (3)
C5—C4—C7—C6	34.1 (4)	C7—C4—O1—C1	−62.5 (3)
C3—C4—C7—C6	171.8 (3)	C2—C1—O1—C4	−49.4 (3)
O1—C4—C7—C8	39.7 (4)	C8—C1—O1—C4	62.7 (3)
C5—C4—C7—C8	156.4 (4)	C5—N1—S1—O2	160.6 (3)
C3—C4—C7—C8	−65.9 (4)	C6—N1—S1—O2	−48.6 (4)
O1—C4—C7—Br1	161.2 (2)	C5—N1—S1—O3	31.1 (4)
C5—C4—C7—Br1	−82.2 (4)	C6—N1—S1—O3	−178.1 (3)
C3—C4—C7—Br1	55.6 (4)	C5—N1—S1—C9	−83.9 (3)
C6—C7—C8—C1	108.8 (4)	C6—N1—S1—C9	66.9 (3)
C4—C7—C8—C1	−2.0 (4)	C10—C9—S1—O2	23.2 (4)
Br1—C7—C8—C1	−121.5 (3)	C14—C9—S1—O2	−160.2 (3)
O1—C1—C8—C7	−35.8 (4)	C10—C9—S1—O3	154.3 (3)
C2—C1—C8—C7	69.5 (4)	C14—C9—S1—O3	−29.1 (4)
C14—C9—C10—C11	−0.6 (6)	C10—C9—S1—N1	−92.0 (3)
S1—C9—C10—C11	176.1 (3)	C14—C9—S1—N1	84.6 (3)
C9—C10—C11—C12	−0.3 (7)		

### Hydrogen-bond geometry (Å, °)

**Table:**

*D*—H···*A*	*D*—H	H···*A*	*D*···*A*	*D*—H···*A*
C6—H6A···O1^i^	0.97	2.50	3.382 (6)	151
C10—H10···O2	0.93	2.59	2.937 (6)	103

Symmetry codes: (i) −*x*+2, *y*+1/2, −*z*+1/2.
